# T-2 toxin Analysis in Poultry and Cattle Feedstuff

**DOI:** 10.17795/jjnpp-13734

**Published:** 2014-04-03

**Authors:** Issa Gholampour Azizi, Masumeh Azarmi, Naser Danesh Pouya, Samaneh Rouhi

**Affiliations:** 1 Department of Veterinary Sciences, Islamic Azad University, Babol Branch, Babol, IR Iran; 2 Department of Microbiology, Cellular and Molecular Research Center, Kurdistan University of Medical Sciences, Sanandaj, IR Iran

**Keywords:** T-2 Toxin, Poultry, Cattle

## Abstract

**Background:**

T-2 toxin is a mycotoxin that is produced by the *Fusarium* fungi. Consumption of food and feed contaminated with T-2 toxin causes diseases in humans and animals.

**Objectives:**

In this study T-2 toxin was analyzed in poultry and cattle feedstuff in cities of Mazandaran province (Babol, Sari, Chalus), Northern Iran.

**Materials and Methods:**

In this study, 90 samples were analyzed for T-2 toxin contamination by the ELISA method.

**Results:**

Out of 60 concentrate and bagasse samples collected from various cities of Mazandaran province, 11.7% and 3.3% were contaminated with T-2 toxin at concentrations > 25 and 50 µg/kg, respectively. For mixed poultry diets, while 10% of the 30 analyzed samples were contaminated with > 25 µg/kg, none of the tested samples contained T-2 toxin at levels > 50 µg/kg.

**Conclusions:**

The results obtained from this study show that poultry and cattle feedstuff can be contaminated with different amounts of T-2 toxin in different conditions and locations. Feedstuff that are contaminated by this toxin cause different diseases in animals; thus, potential transfer of mycotoxins to edible by-products from animals fed mycotoxin-contaminated feeds drives the need to routinely monitor mycotoxins in animal feeds and their components. This is the basis on which effective management of mycotoxins and their effects can be implemented.

## 1. Background

T-2 toxin is a toxic secondary metabolite, which belongs to the trichothecenes group. It is produced by fungi of the following species, *F. acuminatum*, *F. poae *and *F. sporotrichioides*. The basic structure of T-2 toxin is tetracyclic, with a sesquiterpenoid 12, 13 epoxytrichothec-9-ene ring system. This toxin is mainly found in wheat, maize, barley, oats and rye and processed grains (1, 2). Studies have shown that consumption of food and feed contaminated with T-2 toxin causes different diseases in humans and animals. Different animals such as poultry, swine and cattle are at a greater risk due to the consumption of high levels of cereals and oilseeds in the diet (3). Poultry are more sensitive to trichothecenes than ruminants (4). Toxic effects of T-2 toxin in mammals include reduced production and reproduction, dermatonecrosis, gastroenteritis, feed refusal, coagulopathy, immunosuppression and bone marrow depression and destruction in protein synthesis (1). Consumption of feed contaminated with the T-2 toxin at a level of 640 ppb for 20 days causes bloody feces and abomasal and ruminal ulcers, which ultimately lead to death (5). In poultry, the toxic effects of T-2 toxin can be classified as genotoxic and cytotoxic; this toxin can affect the immunomodulatory system, cells of the digestive system and liver, nervous system and skin and can impair poultry performance (6). Some countries have set a guidance value for T-2 toxin in animal feeds. In China, the T-2 toxin limit in animal feed is 0.08 mg/kg. In Canada, feed for swine and poultry can contain up to 1 mg/kg of T-2 toxin (2). However, most countries (such as Russia, Bulgaria, Armenia, and Estonia) have accepted 100 μg/kg as their standard (7). In Iran the standard limit is 25 µg/kg for cattle and poultry feedstuff (8). Various studies have been conducted on cattle and poultry feedstuff for their amount of mycotoxins; Wang et al. reported that of 420 analyzed feedstuff samples in China, the incidence of T-2 toxin, zearalenone and fumonisin B1 was 79.5% (10-735 μg/kg), 85.2% (35-1478 μg/kg) and 96.1% (20-6568 μg/kg), respectively (9). Sokolovic et al. in Croatia showed that of 465 grains and poultry feed samples, T-2 toxin, diacetoxyscirpenol and deoxynivalenol were detected in 16.8%, 27.6% and 41.2% of the samples, respectively (0.05-3.4 mg/kg) (10). Driehuis et al. reported that in the Netherlands of 169 feedstuff samples of dairy cows, T-2 toxin was not detected in any of the samples (11). The aim of this study was to analyze the incidence T-2 toxin in poultry and cattle feedstuff in the Mazandaran province.

## 2. Objectives

Since mycotoxin contamination of feeds results in economic loss, transmission of toxins in the food chain and different diseases in animals and humans, analysis of feedstuffs from the Mazadaran province in Northern Iran for T-2 toxin was necessary.

## 3. Materials and Methods

### 3.1. Sample Collection and Pre-aration

In this study, cattle feedstuff (concentrate and beetroot bagasse) and complete poultry feed samples were randomly collected during winter of 2013, from Sari, Babol and Chalus cities of Mazandaran province, Nothern Iran. For each foodstuff, 10 samples were collected from each city; 60 cattle feedstuff samples were gathered in this order: 20 samples from Babol (10 concentrate, 10 beetroot bagasse), 20 samples from Sari (10 concentrate, 10 beetroot bagasse) and 20 samples from Chalus (10 concentrate, 10 beetroot bagasse). Also 30 mixed poultry feedstuff samples consisting of 10 samples from Babol, 10 samples from Sari and 10 samples from Chalus were gathered, making a total of 90 samples. Samples were then put into sterile plastic bags to be protected from light. Samples were put into sealed sterile plastic bags and transported to a Mycology laboratory where they were ground to fine powder.

### 3.2. Analytical Procedure

T-2 toxin was extracted from samples and analyzed according to the manufacturer's specifications. Accordingly, 20 g of the toxin was weighed and added to a blender containing 100 mL of 70% methanol and blended for three minutes. The extract was allowed to stand for a few minutes and the content was filtered through a Whatman No. 1 filter paper. T-2 toxin contamination in extracts was measured by competitive enzyme-linked immunosorbent assay (ELISA) using the Agraquant T-2 toxin assay kit (RomerLabs, Singapore).

### 3.3. ELISA Procedure

T-2 toxin contamination rate of the samples was measured by competitive enzyme-linked immunosorbent assay (ELISA) using the Agraquant T-2 toxin assay kit (purchased from the Romer Singapore Company). Firstly, we matched the number of samples to the number of wells in the kit. Next, 200 μL of the conjugated solution was added to uncoated-antibody microplate wells. 100 μL of each standard solution (0, 75, 150, 300 and 500 ppb) was diluted in distilled water with a ratio of 1:10, in the test tubes. Then, 100 μL of the above solution was transferred to coated-antibody microplate wells. Toxins in samples and control standards competed with the enzyme conjugate for binding to the solid phase antibody. They were incubated at room temperature (37˚C) and washed after 15 minutes by distilled water with a sprinkler. After the washing step, 100 μL of the enzyme substrate was added to wells and incubated at room temperature for another five minutes. Tetra methylbenzidine/hydrogen peroxide was used as a substrate for color development. After the washing step, a blue color was observed in the wells. Finally, 100 μL of stopping solution was added to stop the reaction and then the blue color turned yellow. The color intensity was inversely proportional to the mycotoxin concentration and measured with the ELISA reader (450-630 nm). Toxin concentration in the samples was compared with standard concentrations and absorption by using a standard curve. Optical density (OD) readings were recorded for each microwell (using either the unmodified OD values or the OD values expressed as a percentage of the OD of the zero (0) standard, a dose-response curve with the five standards was constructed). Since the amount of T-2 in each standard was known, the unknowns could be measured by interpolation from this standard curve. Concentration of T-2 toxin was calculated by analysis of variance (ANOVA) using the SPSS software package (P < 0.05) (according to manufacturer's instruction).

## 4. Results

Of the 20 samples collected from Babol, only 5% showed positive contamination with > 25 µg/kg of T-2 toxin and none of the samples were contaminated with > 50 µg/kg of the T-2 toxin (mean ± SE: 10.40 ± 1.82). Out of 20 samples gathered from Sari, 15% were contaminated with > 25 µg/kg and 5% with > 50 µg/kg (mean ± SE: 12.12 ± 3.05). Also in Chalus 15% of samples were contaminated with > 25 µg/kg while 5% of samples were contaminated with > 50 µg/kg (mean ± SE: 11.48 ± 2.92). Based on the experiments done on the cattle feedstuff samples, gathered from the 3 mentioned cities of the Mazandaran province, we concluded that out of 60 samples, 11.7% and 3.3% were infected with > 25 µg/kg and > 50 µg/kg of the toxin, respectively. The highest contamination level of the T-2 toxin was observed for Sari and Chalus samples. Samples collected from Babol had a lower concentration of toxin compared to samples from Sari and Chalus (Table 1).

**Table 1.** Distribution of T-2 Toxin Contamination in Cattle Feedstuff Samples Based on Cities (n = 60) ^a, b^

Cities	> 25 µg/kg	> 50 µg/kg	Results		Maximum	Minimum
			Mean ± SE	Mean ± SD		
**Babol (n = 20)**	1 (5)	0	10.40 ± 1.82	10.40 ± 8.15	26.1	0.1
**Sari (n = 20)**	3 (15)	1 (5)	12.12 ± 3.05	12.12 ± 13.64	53.5	0.1
**Chalus (n = 20)**	3 (15)	1 (5)	11.48 ± 2.92	11.48 ± 13.06	52.5	0.1
**SUM**	7 (11.7)	2 (3.3)	11.45 ± 1.52	11.45 ± 11.69	53.5	0.1

^a^ Abbreviations: SE, standard error; SUM, sample statistical summary.

^b^ Data are presented as No. (%).

Tests for T-2 toxin contamination in the cattle feedstuffs revealed that 13.3% of 30 concentrate samples had > 25 µg/kg of contamination and 3.3% were contaminated with > 50 µg/kg (mean ± SE: 10.24 ± 2.21). Tests on the beetroot bagasse samples showed that, 10% had > 25 µg/kg and 3.3% had > 50 µg/kg of T-2 toxin infection (mean ± SE: 12.76 ± 2.07). In total, from the 60 samples, 11.7% had > 25 µg/kg and 3.3% had > 50 µg/kg of contamination (mean ± SE: 11.45 ± 1.51) (Table 2).

**Table 2.** Distribution of T-2 Toxin Contamination in Cattle Feedstuff Samples Based on Feedstuff (n = 60) ^a^

Feed Stuff	> 25 µg/kg	> 50 µg/kg	Results		Maximum	Minimum
			Mean ± SE	Mean ± SD		
**Concentrated (n = 30)**	4 (13.3)	1 (3.3)	2.21 ± 10.24	2.21 ± 12.11	52.5	0.1
**Beetroot bagasse (n = 30)**	3 (10)	1 (3.3)	2.07 ± 12.67	2.07 ± 11.34	53.5	0.1
**SUM**	7 (11.7)	2 (3.3)	1.51 ± 11.45	1.51 ± 11.69	53.5	0.1

^a^ Data are presented as No. (%).

Also, of the ten mixed poultry feedstuff samples that were collected from Babol, two (10%) samples showed > 25 µg/kg of T-2 toxin contamination, with this value being higher than the Iran Standard Institute limit (mean ± SE: 13.05 ± 3.43). In Sari, none of the mixed poultry feedstuff samples were contaminated with > 25 µg/kg and > 50 µg/kg of this toxin. Despite the contamination of samples, there was no contamination higher than the standard limit of European Union (EU) and Iran Standard Institute (mean ± SE: 11.03 ± 2.96). Of the ten collected mixed poultry feedstuff samples from Chalus, only one (10%) sample was contaminated with > 25 µg/kg (mean ± SE: 11.20 ± 3.53). Of the 30 poultry samples, three (10%) were contaminated with > 25 µg/kg of T-2 toxin (mean ± SE: 11.76 ± 1.86). > 25 µg/kg of T-2 toxin contamination was observed in Babol and Chalus, respectively. None of the mixed poultry feedstuff samples were contaminated with > 50 µg/kg (Table 3).

**Table 3.** Distribution of T-2 Toxin Contamination in Mixed Poultry Feedstuff Samples Based on Cities (n = 30) ^a, b^

Cities	> 25 µg/kg	> 50 µg/kg	Results		Maximum	Minimum
			Mean ± SE	Mean ± SD		
**Babol (n = 10)**	2 (20)	ND	13.05 ± 3.43	13.05 ± 10.86	27.5	0.1
**Sari (n = 10)**	ND	ND	11.03 ± 2.96	11.03 ± 9.36	24	0.1
**Chalus (n = 10)**	1 (10)	ND	11.20 ± 3.53	11.20 ± 11.6	27.5	0.1
**SUM**	3 (10)	ND	11.76 ± 1.86	11.76 ± 10.16	27.5	0.1

^a^ Abbreviation: ND, no detected.

^b^ Data are presented as No. (%).

## 5. Discussion

T-2 toxin is toxic to humans, mammals, birds, invertebrates, plants and eukaryotic cells. Symptoms of T-2 toxin poisoning depend on the dose and way of exposure (2). Consumption of feed that is contaminated with T-2 toxin at a level of 640 ppb (ppm = 1000 ppb) for 20 days causes bloody feces, abomasal and ruminal ulcers which may lead to death (5). Results of this study, based on all three cities (Babol, Sari and Chalus), showed that of the 60 concentrate and beet bagasse samples, the highest contamination level of T-2 toxin was detected in Sari, and Chalus samples had greater contamination than Babol samples. Out of the 60 concentrate and beet bagasse samples that were tested in our study, the highest contamination level of T-2 toxin was observed in the city of Sari with a maximum level of 53.5 µg/kg and Chalus with a maximum level of 52.5 µg/kg. Therefore, the results of this study revealed that all cattle feedstuff samples had a higher T-2 toxin contamination than the ISIRI’s authorized limits. A study on mixed poultry feedstuff samples showed that the maximum level of this toxin was 27.5 µg/kg, which is again higher than the ISIRI’s standard limit. However, contamination rates were not higher than the standard limit of the EU. Several reports from Iran have revealed the presence of mycotoxins in commodities. In a study carried out in Iran by Riazipour et al. levels of T-2 toxin in 46 grain samples used for human consumption were tested and results showed that all of the tested samples were contaminated with T-2 toxin with a range of 7.9 to 65.9 µg/kg, which is higher than the T-2 toxin concentrations found in our study (7). Despite the contamination of cattle feedstuff samples observed in our study, there was no feed sample with a contamination rate higher than the standard limit of the EU. T-2 toxin Production is predominant in tropical and subtropical regions so amounts of this toxin may be different according to the geographical area. For example, warm and moist weather are favorable for *Fusarium* spp. growth (6, 12). Aksoy et al. in Turkey, using the ELISA method showed that the incidence of T-2 toxin in 40 compound ruminant feed samples was 87.5% (51.61-1023.25 μg/kg) (13). In our study, the T-2 toxin contamination in the diet of cattle showed that of the 60 samples, 11.7% were contaminated with > 25 µg/kg and 3.3% were contaminated with > 50 µg/kg. T-2 toxin production is predominant in tropical and subtropical regions and warm and moist weather (13 % to 22 %) conditions, which favor *Fusarium* spp. infection of plants (6). Also concentrations of different mycotoxins according to geographical areas, seasonal alterations, climatic factors, water activity, moisture and substrate are different (12). Charoenpornsook and Kavisarasai performed a study in Thailand and detected T-2 toxin in all of the ten animal feedstuff samples (mean concentration: 6.91 ppb) (3). However, in our study, of the 60 samples of beetroot bagasse and concentrate, seven were contaminated with > 25 µg/kg and two samples were contaminated with > 50 µg/kg. Kocasari et al. using the ELISA method in 180 dairy cattle, beef cattle, and lamb-calf feeds in Turkey showed that T-2 toxin were found in 85 (47.2 %) samples (3.85-52.36 μg/kg) (14). Cortinovis et al. in Italy reported that of 72 samples of raw materials for equine feed, T-2 toxin was found in 12.3% (12-102 μg/kg) (15). In our study T-2 toxin concentrations of all collected samples for cattle feedstuff ranged from 5 to 0.1 µg/kg, which is less than that reported by Kocasari and Cortinovis. Other environmental factors such as temperature and pH can lead to the difference between the levels of contamination reported by our study and other previous reports (12). T-2 toxin is produced at temperatures between 0˚C and 32˚C and *Fusarium* species cannot survive in low oxygen and low pH. For example, *F. sporotrichioides* has a low optimal temperature (6˚C to 12˚C) for T-2 toxin production and can produce this mycotoxin during overwintering, under a snow cover in the field and/or during storage (6). Labuda et al. in Slovakia, using gas chromatography electron capture detection (GC-ECD) showed that of 50 samples of poultry feed mixtures, 45 (90%) samples were contaminated with T-2 toxin (1-130 µg/kg) and a contamination level higher than our study (0.1-27.5 µg/kg) (16). Since some mycotoxins can be produced by more than one mold species while some fungal species produce several types of mycotoxins and due to the presence of different species spores and toxin-producer species in different environments, amount of toxin in samples may be different in our study environment compared to previous studies (12). Grajewski et al. in Poland studied 1255 samples of cereal, corn grains and feedstuffs (silages, mixed feeds) by using HPLC methods with fluorescent, UV and MS/MS detection and showed that the highest average concentration of trichothecenes (NIV, T-2 and HT-2 toxins) was < 5.0-139 ng/g (17). Our study was conducted using the competitive enzyme-linked immunosorbent assay (ELISA). Difference in analytical methods may also cause disagreement among the results and contamination level of samples by mycotoxin (18). Greco et al. in Argentina studied rabbit and chinchilla feeds and showed that T-2 toxin was recovered from 98% of samples (19). In our report, none of the mixed poultry feedstuff samples were contaminated with > 50 µg/kg and contamination for > 50 µg/kg was equal with concentrate and beetroot bagasse samples. Mycotoxin production is associated with hydration of feedstuffs, improper storage, low feedstuff quality, inadequate feeding conditions, hygienic condition and workers health in the environment. Thus physical properties of samples, characteristics of the environment and sample collection methods can be effective on the levels of contamination (6, 12, 14, 18). Selection of feedstuffs with high-quality and prevention of mycotoxins uptake by use of food additives reduce mycotoxins while use of appropriate methods for agriculture and storage and fungicides reduce pollution by fungi and mycotoxins (20). In Northern regions of Iran, the climate is suitable for fungi growth compared to other parts of the country (the weather is humid and mild in this part of Iran) (21). Thus, detection of mycotoxins and development of better strategies for management and reduction of fungi growth and mycotoxins, in this area are crucial.
